# Control and elimination of rabies in Croatia

**DOI:** 10.1371/journal.pone.0204115

**Published:** 2018-09-20

**Authors:** Tomislav Bedeković, Ivana Lohman Janković, Ivana Šimić, Nina Krešić, Ivana Lojkić, Ivica Sučec, Emmanuelle Robardet, Florence Cliquet

**Affiliations:** 1 Croatian Veterinary Institute, Zagreb, Croatia; 2 Veterinary Directorate, Zagreb, Croatia; 3 Nancy OIE/WHO/EU Laboratory for Rabies and Wildlife, French Agency for Food, Environmental and Occupational Health &Safety, Malzéville, France; US Geological Survey, UNITED STATES

## Abstract

Despite the implementation of control measures (preventive dog vaccination), rabies has become endemic in Croatia, with red foxes being the main reservoir species. Oral rabies vaccination (ORV) campaigns supported by the European Commission have been conducted twice a year since the spring of 2011. The first campaigns were limited to the northern and eastern parts of the country, and from the autumn of 2012, the program was extended to the entire country. The Lysvulpen vaccine containing the SAD Bern strain was used for ORV. Following the vaccination campaigns, the number of rabies cases decreased, and the last positive case was recorded in February 2014. The bait uptake ranged from 24.86% to 84.62% and the immunisation rate from 11.24% to 35.64%.

## Introduction

Rabies is a zoonotic disease still present in Europe; the red fox (*Vulpesvulpes*) is the virus’s main reservoir and vector [1]. Epidemiological data have confirmed the presence of rabies in wildlife since 1977 (except on the Croatian islands) [2]; thus, it presents a major veterinary and public health threat. Between 1977 and 2010, a total of 108,190 domestic and wild animals were sent to the National Reference Laboratory for Rabies at the Croatian Veterinary Institute for rabies testing. Of these, 16,723 (15.5%) showed positive rabies findings (unpublished). Foxes were the most commonly tested of all animals (60.9%). Of 65, 887 tested foxes, 23.6% had positive rabies findings [2]. The last human rabies case was recorded in 1964 [3,4]. The fact that no further human rabies cases have been recorded is mainly attributed to the forceful implementation of prophylactic measures, such as quarantine, registration, and mandatory vaccination of dogs; capture and culling of stray dogs and cats; preventive vaccination of individuals in high-risk occupational groups; strict application of post-exposure prophylaxis; educational campaigns; and close cooperation between veterinary and public health institutions. However, these measures are insufficient to eliminate rabies in Croatia because the virus still thrives in wild animals. To improve the animal health situation in Croatia, in particular concerning those diseases that continue to be a threat to European Union (EU) Member States, such as rabies, Croatia started oral rabies vaccination (ORV) of foxes in the spring of 2011 with the support of the EU through the Instrument for Pre-Accession assistance (IPA) [5]. A modified live virus rabies vaccine using the SAD Bern strain was selected for oral vaccination. This strain is not recommended by the WHO [6] because it is potentially pathogenic for several rodent species as well as wild and domestic carnivores, irrespective of the inoculation route [7]. However, at the time the ORV program was initiated, this was the only vaccine registered in Croatia. Three vaccination campaigns (spring and autumn of 2011 and spring of 2012) were conducted in the northern part of Croatia (overall coverage area: 35,000 km^2^). Campaigns beginning in the autumn of 2012 then spring and autumn of 2013 were expanded to the entire mainland territory. In July 2013, Croatia joined the EU. Starting in the spring of 2014, the IPA project was ended, and ORV continued with the financial support of the European Commission [8]. The objective of this study was to present a comprehensive description of the efforts it took to eliminate rabies in Croatia, using serological results, bait uptake, and surveillance data to monitor progress. Oral vaccination efficacy was assessed using the incidence of rabies in the country, proportion of the fox population having consumed the baits (revealed using an oxytetracycline biomarker incorporated to the bait matrix), and rabies immunisation rate [9].

## Material and methods

### Oral vaccination strategy

The oral vaccination strategy was organised in accordance with the guidelines and conclusions of the European Commission [1,10]. The Republic of Croatia is located in south eastern Europe. The entire territory covers 87,967 km^2^ and is bordered by Bosnia and Herzegovina and Serbia in the east, Slovenia in the west, Hungary in the north, and Montenegro and the Adriatic Sea in the south. Croatia’s territorial waters encompass 18,981 km^2^, and its internal waters cover an additional 12,498 km^2^. The affected area corresponds to the mainland surface area, i.e. around 56,542 km^2^. The contractor for bait distribution was selected using a public tendering procedure. The vaccine baits used (Lysvulpen, Biovetaa.s. Ivanovice na Hané, Czech Republic) contained a modified live virus SAD Berne MSV Bio 10 strain (http://www.bioveta.eu/en/products/veterinary-products/lysvulpen-por-ad-us-vet-1.html). Oxytetracycline was used as a biological marker incorporated in the bait matrix (150 mg per bait) to assess bait consumption [1]. Vaccine baits were transported while refrigerated from the Czech Republic to Croatia in trucks (-20°C) and stored at -20°C prior to use. Before each vaccination campaign, ten baits from each batch of vaccine were titrated by the National Reference Laboratory for Rabies at the Croatian Veterinary Institute according to the instructions of the EU Reference Laboratory for Rabies (Nancy, France). According to the national legislation in Croatia, the vaccine should have a titre between 10^7.0^ and 10^8.0^ tissue culture infective doses _50_/ml. As of 2011, baits were distributed twice a year: in the spring (April to June) and autumn (October to November) [1,10]. From storage to delivery in the field, baits were transported in vehicles containing the equipment needed to keep the temperature at -20°C. At every airport, cold storage facilities (-20°C) were provided for the number of baits needed for a one-day flight (refrigerated trucks or freezer). Baits were distributed along parallel flight lines 500 m apart [1,10]. They were dropped automatically using a small fixed-wing aircraft (Cessna 172 with a GPS system for recording the exact location of dropped baits) flying at an altitude of 100–150 m and a speed of 100–150 km/h. Twenty-five baits were distributed per km^2^ with approximately 80 m between each bait. No baits were dropped over rivers, lakes, river channels, streams, motorways, highways or other roads, urban areas (cities, villages, public areas, beaches, and campsites), or mountains over 1,300 m above sea level. The distribution of baits in 2011 was initially confined to 35,000 km^2^ (16 counties) in the northern part of the country. From the autumn of 2012, the coverage area was extended to the 56,542 km^2^comprisingthe total area of Croatia, including all 21 counties. Approximately 875,000 baits were distributed per campaign during the first three campaigns, and since the autumn of 2012, 1,413,550 baits have been dropped per campaign. Because no positive cases have ever been recorded on the Croatian islands (3,399 km^2^), no baits were distributed on the Adriatic islands. The ORV program in Croatia was organised with neighbouring countries involved in a program designed to control and eradicate rabies in western Balkan countries [11].

### Ethical issues

The animals studied in the framework of surveillance and monitoring schemes originating from the field died of natural causes or during the vaccination/hunting program developed and initiated by competent authorities (Veterinary Directorate). The sampling procedures were performed in compliance with the country’s own legislation and the recommendations of international institutions [12,13]. In Europe, such procedures do not require any specific ethical approval as hunting plans are organised by national authorities in the framework of disease control programs.

### Evaluation of vaccination efficacy

Vaccination efficacy was evaluated using rabies incidence (surveillance), bait uptake, and humoral anti-rabies immune response (monitoring of ORV) [9].

#### Rabies surveillance

Rabies has been a notifiable disease in Croatia since 1977. Rabies surveillance complies with the recommendations of international organisations [1,6,10] and is based on the laboratory testing of wild and domestic animals sampled countrywide. All suspect animals and those found dead must be reported to veterinarians and submitted to the National Reference Laboratory or Official Regional Laboratories for Rabies. Rabies was diagnosed by detecting the rabies virus (RABV) using the WHO/OIE reference test, i.e. the fluorescent antibody test in the brain material [14]. In the event of contact between rabies-suspected animals and humans, the samples were also tested using RT-PCR to avoid potentially false negative results [15]. Randomly selected samples from rabies-positive animals between 2008 and 2010 have already been tested [16]. This study reports sequencing data on 31 randomly selected positive cases from surveillance detected between 2011 and 2014. Thirty-one further positive samples were selected for phylogenetic analysis as a part of monitoring from 2011 and 2012 to differentiate animal rabies cases due to vaccine-associated infections from cases due to field strains and to assess similarities with strains in other countries.

#### Phylogenetic analysis

For the extraction of RNA and RT-PCR, 10% (w/v) brain material suspensions were prepared using Dulbecco's Modified Eagles Medium. RNA was automatically extracted from the supernatant of centrifuged suspensions using the iPrep Pure Link Virus kit and iPrep Instrument (Invitrogen) according to the manufacturer’s instructions. For partial sequencing, a one-step RT-PCR was performed with 5 μl of RNA and 2 pmol of pan-lyssavirus primer JW12 (5’-ATGTAACACCYCTACAATG-3’) and JW6DPL (5’-CAATTCGCACACATTTTGTG-3’) as described [15] using Superscript III with Platinum Taq polymerase (Invitrogen, Carlsbad, USA). Amplified products (606 bp) were visualised using agarose gel electrophoresis and subsequently purified using Exosap (USB, Staufen, Germany). Purified PCR products were sequenced in both directions by Macrogen Inc. (Seoul, Korea). The nucleotide sequences generated in this study were subsequently submitted to GenBank and assigned the following accession numbers: KX929097—KX929159. The sequences obtained were assembled and edited using MEGA version 6 [17]. A 367 bp part (nucleotides 75–441 of the challenge virus standard reference strain; GenBank accession no. D42112) was used for further analysis. A phylogenetic tree of classic RABV nucleoprotein sequences was constructed using the Kimura2-parameter neighbor joining method with MEGA version 6 and rooted with the RABV sequence present in the Lysvulpen vaccine. A phylogenetic tree was established among 63 Croatian RABV N sequences (2013–2014), two previously published Croatian sequences with GenBank accession numbers GU134624 and JF683628, and nine representative sequences from GenBank.

#### Enhanced ORV monitoring

The objective of monitoring is to assess the effectiveness of the vaccination campaigns by determining the bait uptake and seroconversion rates in the target animals sampled in vaccinated areas [9]. To obtain a sufficient number of samples, authorised hunter associations and official veterinary stations were offered a financial incentive for hunted animals in the field (through a budget provided by competent national authorities). The fox carcasses were delivered by hunters to official veterinary stations and consequently sent to the laboratory for testing, together with an accompanying document. At the beginning of the ORV program in 2011 and 2012, the carcasses of eight foxes per 100 km^2^were collected annually from hunting activities. Since 2013, only four foxes per 100 km^2^ were collected annually as previously recommended [6,9]. The foxes collected in the framework of ORV monitoring were also analysed for rabies as recommended [9] using the same techniques as those used for rabies surveillance. In 2017, carcasses of jackals were also included in the monitoring.

#### Bait uptake

A canine tooth with alveolar bone tissue was isolated from the lower jaw of the sampled foxes to assess the presence of oxytetracycline using a fluorescence microscope as previously described [18]. The age of the oxytetracycline-positive animals was also determined as previously described [19]. Based on the results, the foxes and jackals were classified as younger or older than 12 months [20].

#### Rabies immunity

The animals were brought to the veterinary services by the authorised hunters involved in the sampling scheme throughout the year; thus, blood was not collected immediately after the animals’ death. This resulted in serum samples of a poor quality. A modified FAVN test [21] was developed and validated [22] for testing poor quality sera. Named the mFAVN test, it was used for testing rabies antibodies in muscle juices. Two indirect ELISA kits were also used: the Platelia rabies II kit (Bio-Rad, France) and the BioPro Rabies ELISA Ab kit (BioPro, Czech Republic) adapted and validated on muscle juices or thoracic liquids [23]. The first kit was used to evaluate the results of the first campaign (spring of2011), while the second was used in all further campaigns (autumn of 2011 to 2017) according to the recommendations of the EU Reference Laboratory for Rabies [24]. The threshold of positivity for ELISA was set in accordance with the manufacturer’s recommendations. When the mFAVN test was used, the threshold of antibody detection was set at 0.1 IU/ml as described previously [22].

### Statistical analysis

STATA 10 (Stata Press, College station, Texas, USA) was used to compute the 95% confidence intervals for proportions (i.e. proportion of bait uptake and seroconversion) and produce graphs; the Z-test using STATA 13.1 (Stata Press) was used to perform the statistical analyses.

## Results

### Rabies incidence

**Fig 1:**
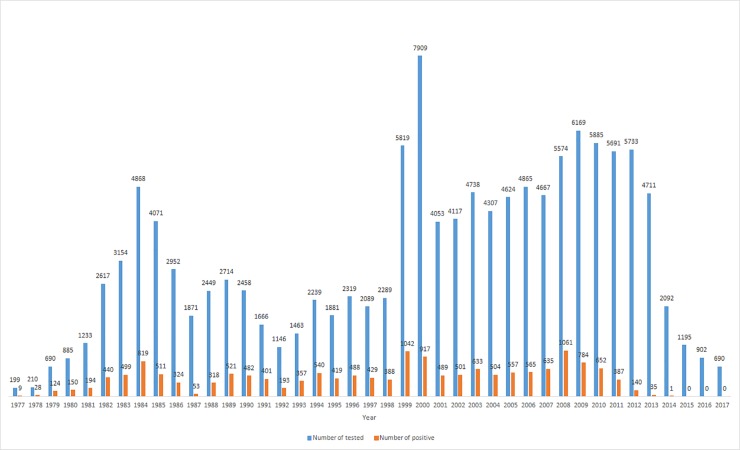
Total rabies-tested animals and rabies cases in Croatia from 1977 to 2017.

Between 1977 and 2010, 108,190 domestic and wild animals were tested for rabies, of which 16,723 (15.5%) were found to have positive findings (Fig 1). Red foxes were the most frequently tested species (60.9%). Of all the tested foxes (64,900), 23.6% had positive findings for the RABV. The most commonly infected species after red foxes were dogs (2.7%) and cats (2.4%). The number of positive cases has dropped drastically since 2011 (Fig 1) as a result of the oral vaccination of foxes against rabies in the northern, central, and eastern parts of the country. After extending ORV to the entire territory, the number of positive cases rapidly decreased, and in 2013, only 35 positive cases were detected, of which 33 involved foxes, one a dog, and one a horse. In the same year, the majority of the positive cases were recorded near the city of Zagreb (32 rabies-positive foxes) and in the southern part of the country (one dog, one horse, and one fox). The last positive case was recorded in February 2014 in a fox in Zagreb county (Table 1).

**Table 1:** Number of rabies cases in the foxes and jackals from 2011 to 2017.

Year	Number of tested animals		Number of rabies-positive animals	
	Routine surveillance	Enhanced monitoring	Routine surveillance	Enhanced monitoring
**2011**	3597	740	329	22
**2012**	3748	1492	118	9
**2013**	3376	2042	33	0
**2014**	1024	3151	1	0
**2015**	447	5692	0	0
**2016**	495	1636	0	0
**2017**	315	874^1^	0	0

^1^Includes 30 jackals.

### Phylogenetic analysis

**Fig 2:**
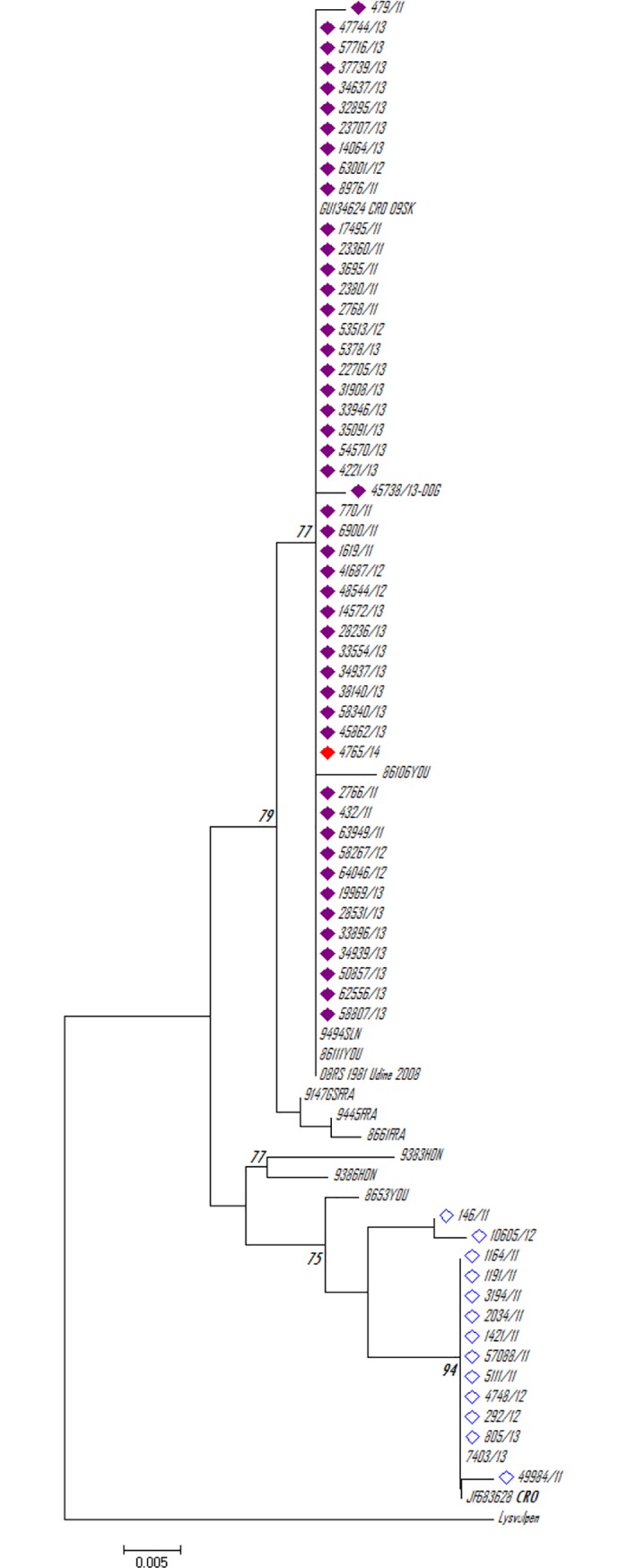
Phylogenetic analysis of the partial N gene of Croatian rabies virus (RABV) from 2011 to 2017, together with the representative sequences from GenBank. The tree was constructed using the neighbor-joining method and rooted with the RABV sequence present in the Lysvulpen vaccine. The analysis included 367 nucleotide positions. Bootstrap values below 70% are not shown. The scale bar indicates the number of nucleotide substitutions per site. All RABVs belonging to the formerly established western European group are marked with a purple lozenge, while those belonging to the eastern European group are marked with a blue lozenge. The sequence from the last positive Croatian case is marked with a red lozenge.

After a partial sequencing of the N gene, 31 isolates randomly selected from 2013 and the last case from 2014 were confirmed as classic RABV. Twenty-nine samples from 2013 were clustered with the formerly established western European (WE) group, while the rest of the isolates (N = 2) were identical to the eastern European (EE) group. Isolate 4765/14 (the last rabies case in 2014) was grouped with the WE strains (Fig 2). The foxes collected by hunters in the framework of enhanced monitoring from2011 to2017 were also analysed for rabies. Partial N gene sequencing of these 31 isolates confirmed the presence of a field strain of classic RABV in all the samples. No vaccine-strain virus was found; however, vaccine-induced rabies cannot be ruled out, since lower than 10% of the positive samples were characterised.

### Bait uptake

Between 2011 and 2017, 14,223 samples were tested for oxytetracycline (Table 2). The age determination data for 2015 and 2016 are not shown because the age was not determined from January to March 2015 and from June to December 2016.

**Table 2:** Bait uptake and oxytetracycline positivity in the foxes (2011–2017) and jackals (2017).

Year	Total tested	Positive % (95%CI)	Tested animals >1 year of age	Positive (95%CI)	Tested animals <1 year of age	Positive % (95%CI)
2011	740	24.86 (21.89–28.10)	302	18.87 (14.86–23.67)	438	29.00 (24.94–33.41)
2012	1489	57.21 (54.69–59.71)	784	71.56 (68.30–74.60)	705	41.27 (37.70–44.95)
2013	2024	74.85 (72.92–76.69)	1109	86.56 (84.43–88.45)	915	60.76 (57.56–63.88)
2014	3054	72.23 (70.62–73.79)	2395	71.69 (69.85–73.46)	659	74.20 (70.73–77.40)
2015	5282	82.99 (81.98–84.00)				
2016	1634	84.70 (82.95–86.45)				
2017	841	75.50 (72.49–78.29)	658	85.41 (82.51–87.90)	183	39.89 (33.07–47.12)
Jackals	30	90.00 (74.38–96.54)	28	96.42 (82.29–99.37)	2	0.00 (0.00–0.65)

The annual number of oxytetracycline-positive foxes over the 2011–2016 period increased from 24.86% to 84.70% (Table 2); that over the 2011–2014 period ranged from 18.87% to 86.47% in the foxes older than 1 year and from 29% to 74.20% in the foxes younger than 1 year (Table 2). The differences in the bait uptake between the adult (>1 year of age) and juvenile (<1 year of age) foxes were significant in 2011, 2012, and 2013 (p<0.01). In 2014, the bait uptake between the adult and juvenile foxes was not significantly different (p = 0.2026). The overall percentage of positive samples from 2011 to 2014 was significantly higher in the adult foxes (71.76%) than in the juvenile foxes (53.84%) (p<0.01). In 2017, the overall percentage of oxytetracycline-positive foxes decreased to 75.50%. Despite the limited number of samples (N = 30), the results of testing in jackals showed a high percentage of bait uptake (90.00%).

### Humoral response

The results for the rabies antibodies in the sampled foxes are shown in Table 3. The annual rates of rabies antibody levels in the foxes during the 2011–2016 period evaluated using the mFAVN test ranged from 18.82% to 34.49% (Table 3). Using ELISA, the results ranged from 3.52% to 37.28%. The differences in the seropositivity obtained between the mFAVN test and ELISA were significant in 2011 and 2012 (p<0.01) but neither in 2013 (p = 0.083) nor 2014 (p = 0.483). Data in 2017 should be considered very carefully and isolated because the number of tested samples was insufficient (only 774 foxes were tested instead of the planned number of 2,200).

**Table 3:** Seroconversion rates in the foxes (2011–2017) and jackals (2017).

Year	Number of tested animals		Seropositive (%)	
	ELISA	mFAVN test	ELISA (95% CI)	mFAVN test (95% CI)
**2011**	682	611	3.52 (2.13–4.91)	18.82 (15.71–21.93)
**2012**	1400	1293	37.28 (34.75–39.82)	27.68 (25.25–30.13)
**2013**	1593	1800	21.34 (19.32–23.36)	23.83 (21.86–25.80)
**2014**	951	2824	23.76 (21.05–26.47)	25.24 (23.64–26.85)
**2015**		4339		34.49 (33.07–35.91)
**2016**		1339		23.83 (21.86–25.80)
**2017**		774		11.24 (9.20–13.66)
**Jackals**		30		20.00 9.51–37.31

The overall percentages of positive samples were significantly higher in the foxes older than 1 year (27.22% tested using ELISA and 26.71% using the mFAVN test) than in the foxes younger than 1 year (20.24% tested using ELISA and 22.41% using the mFAVN test) (p<0.01). The differences in the seroconversion rates between the adult and juvenile foxes tested using both the mFAVN test and ELISA were significant in 2012 and 2013 (p<0.01) but not in 2011 (p = 0.928) or 2014 (p = 0.0542). Data in 2017 should be considered very carefully and isolated because the number of tested samples was insufficient (only 774 foxes were tested instead of the planned number of 2,200). In 2017, the percentages of rabies antibody levels assessed using the mFAVN test were 14.40% in the foxes older than 1 year and 5.88% in the foxes younger than 1 year. Conversely, the percentages of rabies antibody levels assessed using the mFAVN test were 21.42% in the jackals older than 1 year and 50.00% in the jackals younger than 1 year (only two jackals were tested).

## Discussion

In the EU, wildlife rabies has been controlled or even eliminated through the implementation of ORV programs [25]. Economic costs have been the main obstacle to the initiation of such programs in several countries [26]. ORV field trials have been conducted in Croatia along the Istria peninsula and borders with Slovenia as early as 1991 [27], but have been interrupted owing to financial constraints. A nationwide ORV program was implemented in 2011 with the financial support (€4,629,025.34) of the EU [5] through the IPA 2008 and IPA 2010 projects. This ORV project was one of the two components of the disease control programs initiated simultaneously in ‘western Balkan’ countries to eradicate rabies in this region [5]. The main objective of this study was to evaluate the efficacy of the ORV program and the impact of ORV on rabies wildlife reservoir species.

In Croatia, rabies has been a notifiable endemic disease since 1977, with the red fox as the only reservoir species. The implementation of an effective passive surveillance network for collecting any suspect wild or domestic animal, animals found dead, and those to which humans have been exposed [9,13] was established throughout Croatia. Before the start of ORV, around 15% of all tested foxes had positive findings for the RABV on a yearly basis [2]. Taking into account the number of tested samples as a part of the surveillance program (around 3,500 foxes a year), which involved some of the highest numbers of tested foxes in the EU [28], these data confirmed the endemic form of rabies. The rabies incidence has rapidly decreased since 2011, following the implementation of the ORV programs. After ORV was extended to the entire Croatian mainland territory from the autumn of 2012 onwards, the incidence of rabies dropped dramatically, with the last positive case being recorded in February 2014. The persistence of rabies in Zagreb county in 2013 was attributed to areas not fully covered by bait distribution, since they were too close to the Croatian capital. The time from the start of the campaigns to the time when no positive cases were detected was short and comparable to that reported in the Baltic countries [29]. According to results from Estonia, it took three years from the start of ORV implementation to the time when no more positive cases were detected [30]. The situation in Croatia regarding rabies epidemiology and reservoirs cannot be compared to that in Baltic countries because, in Croatia, the main reservoirs of the RABV are red foxes. However, we support the hypothesis that an efficient rabies control program may be conducted by strictly following the different practical recommendations put forward by the European Commission [9,13,31] and WHO [6] every year, particularly those concerning rabies surveillance and ORV implementation. Our results also support those of WE countries, which show that the time required to eliminate rabies through ORV campaigns is not correlated with the number of cases in infected areas [32] or with the size of the infected area.

A previous study has shown that the decrease in the rabies incidence may be connected with the vaccine used [33]. When the efficiency of different vaccines (SAD B19, SAG, and V-RG vaccines) was compared in the field, the results demonstrated a faster and more durable decrease in the rabies incidence with the use of SAG and V-RG vaccine baits [33]. The SAD Bern vaccine used in Croatia was not tested in this study because it was not marketed at that time. However, there was no difference in the efficiency with regards to the time taken to reduce the rabies incidence in Croatia compared with that in Estonia, where the SAG2 vaccine was used. Furthermore, the difference in the bait density (25 baits/km^2^ in Croatia compared with 20 baits/km^2^ in Estonia) could not explain the similar or even greater efficiency in decreasing the rabies incidence in Croatia compared with that in Estonia; a spatial simulation showed that a higher bait density (over 20 baits/km^2^) did not reduce the number of under-baited fox groups and had no beneficial effect on the success of ORV and only wasted resources [34]. A density of 25 baits/km^2^ was selected in Croatia owing to the temperature variations during spring and autumn, taking into account the bait stability data on the SAD Bern vaccine when exposed to temperatures higher than 40°C [13]. It was previously documented that the SAD B19 vaccine can cause side effects [7,29,35,36]. No vaccine-induced rabies cases were detected in Croatia. Phylogenetic analysis of the RABV partial N gene nucleotide sequences undertaken at the beginning of ORV revealed that all the isolates belonged to the classic field RABV strain. There were differences in the representation of formerly established phylogenetic lineages. In 2011 and 2012, there were equal numbers of WE- and EE-grouped isolates, a finding similar to that of our previous phylogenetic analysis of the RABV from domestic and wild animals between 2008 and 2010 [16]. In 2013, the majority of the isolates (N = 29) belonged to the WE phylogenetic group. This is not surprising since the isolates originated from Zagreb county and the southern parts of Croatia. Our previous results showed that WE isolates are exclusively found in the southern and western regions; in central regions along the Save River, both lineages are present [16].

As a part of ORV campaign monitoring, vaccine bait consumption and antibody response were checked in the foxes sampled in vaccinated areas. During the campaigns, the number of samples from the monitoring program gradually increased from 740 in 2011 to 4,775 in 2015. International organisations [6,9] recommend that four foxes should be tested per 100 km^2^, which means that around 2,240 foxes should be tested annually as a part of the Croatian monitoring program. This number was almost achieved in 2014 and 2016 as a consequence of improvement in the coordination between Veterinary Directorate and laboratory with hunting associations. The lower number of foxes tested during the first few years was linked to the campaign organisation, as each hunting association was allowed to collect a limited number of foxes. This restriction was abolished in 2013; thus, there was an increase in the number of foxes shot. However, the smaller number of foxes shot before 2013 probably contributed to the better results obtained in 2012 because it could increase the number of older foxes, which are more likely to consume the baits [37]. The lower number of foxes tested in 2017 was directly connected with the staffing problem in Veterinary Directorate caused by an election. In our study, the bait uptake in the first four years reached 74% and even 84.70% in 2016. In 2017, the bait uptake was 75.50% in the foxes and 90% in the jackals. In 2016, the population of jackals in Croatia increased, and they were also included in the monitoring. Although the number of samples was limited, the obtained results suggest that the baits are very attractive for jackals and that they should be considered as a competitor for bait consumption. In cases of significant increases in jackal populations, the number of baits available to foxes may be decreased, which could have an impact on ORV efficiency. Furthermore, the vaccination responses could be associated with fox and jackal density and bait delivery in open areas. In high fox density habitat, bait uptake might be somewhat compromised as other food and prey options for foxes are abundant. Similarly, compromises of bait uptake probably may be occurred in open areas as such areas are less frequently used by foxes. The data about fox densities per square kilometre are very limited and non-consistent but they showed that the number of foxes per km^2^ increased (0.38 km^2^ in 2011 compared with 0.6 km^2^ in 2016). According to recent studies, the bait uptake could be as high as 80% and can reach 93% [29,30]. The observed reasons for the lower bait uptake in the first four years of ORV was the higher number of juvenile foxes tested. However, the increase in the bait uptake observed every year clearly demonstrates that the strategy was efficient. The number of oxytetracycline-positive samples was higher in the adult foxes than in the juvenile foxes, similar to data from recent studies [29,30], with the exception of the results in 2014. In this year, most of the juvenile foxes were shot during autumn (not after the spring campaign or some weeks after their birth); they had more opportunities to consume the baits during the autumn campaigns as adults. For example, monitoring was applied to juvenile foxes sampled a few weeks after ORV during spring and autumn in France; the mean percentages of the bait uptake were 63% after the spring campaigns and 79% after the autumn campaigns [38].

The immunological response to rabies following ORV is a complicated issue to discuss because different techniques are used for the evaluation, as previously discussed [29]. The humoural response is often assessed using ELISA or virus neutralisation tests [29]. At the beginning of the campaigns, the ELISA kit produced by Bio-Rad (Platelia Rabies II kit) was used [39]. However, a study [24] demonstrated the test’s low sensitivity (79%). Indeed, using this ELISA kit with a positivity threshold of 0.5 EU/ml, the seroconversion rates were very low (3.5% using ELISA versus 18.9% using the mFAVN test). Since 2012 and following the recommendation of the Rabies EURL, a new ELISA kit has been used (BioPro) [39]. Using this kit, the correlations between the results obtained using ELISA and the mFAVN test significantly improved. It should be underlined that the sample quality was very poor, as the animals were collected in the field and transported to the laboratory in charge of sampling blood. Low-quality samples may provide false positive results [40] and cause cytotoxic effects when a virus neutralisation test is applied [41]. From 2011 to 2014, both the mFAVN test and ELISA were used to evaluate the immunological response in foxes. When the mFAVN test was used, the term ‘positivity’ was considered to describe the detection of antibodies (not the 0.5 IU/ml titre) [23]. Between 2015 and 2017, only the mFAVN test was used. The highest percentage of seropositive foxes was recorded in 2015. In 2015, most of the foxes were shot during autumn, in contrast with 2016 when the seropositivity in the foxes was lower with most foxes shot during spring. In 2017, the seropositivity rate was the lowest, with only 11.24% of foxes having specific antibodies. The ORV program was interrupted in 2017, with a decrease in the number of samples because of poor coordination of the competent authority (Veterinary Directorate) caused with the parliamentary election in Croatia. Twenty percent of tested jackals showed positive findings for specific antibodies, suggesting that in the future, the jackal population should also be considered during the planning of ORV campaigns.

Croatia has a very long border with neighbouring countries (2374.9 km), and all the rabies control actions in those countries will have a major impact on the rabies situation in Croatia. Of all the neighbouring countries, only Slovenia is currently officially rabies free[42]. In Hungary and Serbia, rabies is still present with few cases reported each year [43]. The results of the ORV campaigns from the neighbouring countries (Serbia and Bosnia and Herzegovina) show results similar to those in Croatia. In Serbia, the seroprevalence in 2013 was 20.11%, and the bait uptake was 62.60% [44]. In Bosnia and Herzegovina, the bait uptake was 70–80% [45]. However, the most important indicator of the effectiveness of ORV campaigns is the number of positive cases. As the longest border of Croatia is with Bosnia and Herzegovina (1011 km), it is essential that this country and other western Balkan countries continue their rabies control efforts to become rabies free.

### Conclusion

Croatia initiated oral vaccination programs in the spring of 2011 in the northern part of the country. The program was then extended to the entire country from 2012, which eliminated rabies throughout in a short period (32 months), with the last case being recorded in February 2014. The country could be considered rabies free according to the OIE definition (two consecutive years of continuous surveillance after the last case without any more positive cases). However, rabies is still present in several bordering countries, and it is crucial that all neighbouring countries participate in rabies control and elimination programs. These results show the extreme usefulness and effectiveness of the ORV program for the countries where it is implemented.
